# Evolution and Future Directions in Herbicide-Resistant Crop Development and Weed Management

**DOI:** 10.1186/s12284-025-00869-2

**Published:** 2025-11-25

**Authors:** Xiaodong Hou, Yuwen Yang, Qing Liu, Baolong Zhang

**Affiliations:** 1 Provincial Key Laboratory of Agrobiology and Institute of Germplasm Resources and Biotechnology, Jiangsu Academy of Agricultural Sciences, Nanjing, 210014 Jiangsu China; 2 Zhongshan Biological Breeding Laboratory, Nanjing, 210014 Jiangsu China; 3 College of Agriculture, Yangzhou University, Yangzhou, 225009 Jiangsu China

**Keywords:** Herbicide resistance, Genetic engineering, Integrated weed management, Harvest weed seed control, Site-specific weed management

## Abstract

The persistent challenge of weed management in agriculture has been profoundly influenced by the development and adoption of herbicide-resistant (HR) crops. This review examines the historical evolution, current dynamics, and future directions of HR crop development and integrated weed management (IWM) strategies, providing an in-depth analysis of the field. We trace the advent of herbicide resistance in crops, highlighting the genetic and biotechnological advancements that have facilitated the development of crops resistant to various herbicidal modes of action. Concurrently, we explore the biological mechanisms of herbicide resistance in weeds and their implications for agricultural practices and herbicide effectiveness. A significant focus is placed on the renewed efforts in herbicide discovery, highlighting the challenges faced and the innovative approaches being explored, including natural product research and advanced molecular techniques. As the agronomic landscape evolves, the review emphasizes the escalating importance of IWM, presenting it as a multifaceted approach that integrates chemical, cultural, and mechanical strategies to sustainably manage weed populations. Moreover, the review highlights the emergence of non-chemical control measures, such as harvest weed seed control (HWSC) and breeding weed-competitive cultivars, underscoring their role in a comprehensive weed management strategy. The advent of site-specific weed management (SSWM) and its potential to revolutionize weed control practices are critically analysed, discussing the integration of cutting-edge technologies in precision agriculture. Looking forward, we contemplate the challenges and policy implications associated with the widespread adoption of HR crops and IWM practices, emphasizing the necessity for well-informed regulatory frameworks to ensure agricultural sustainability.

## Background

**Fig. 1:**
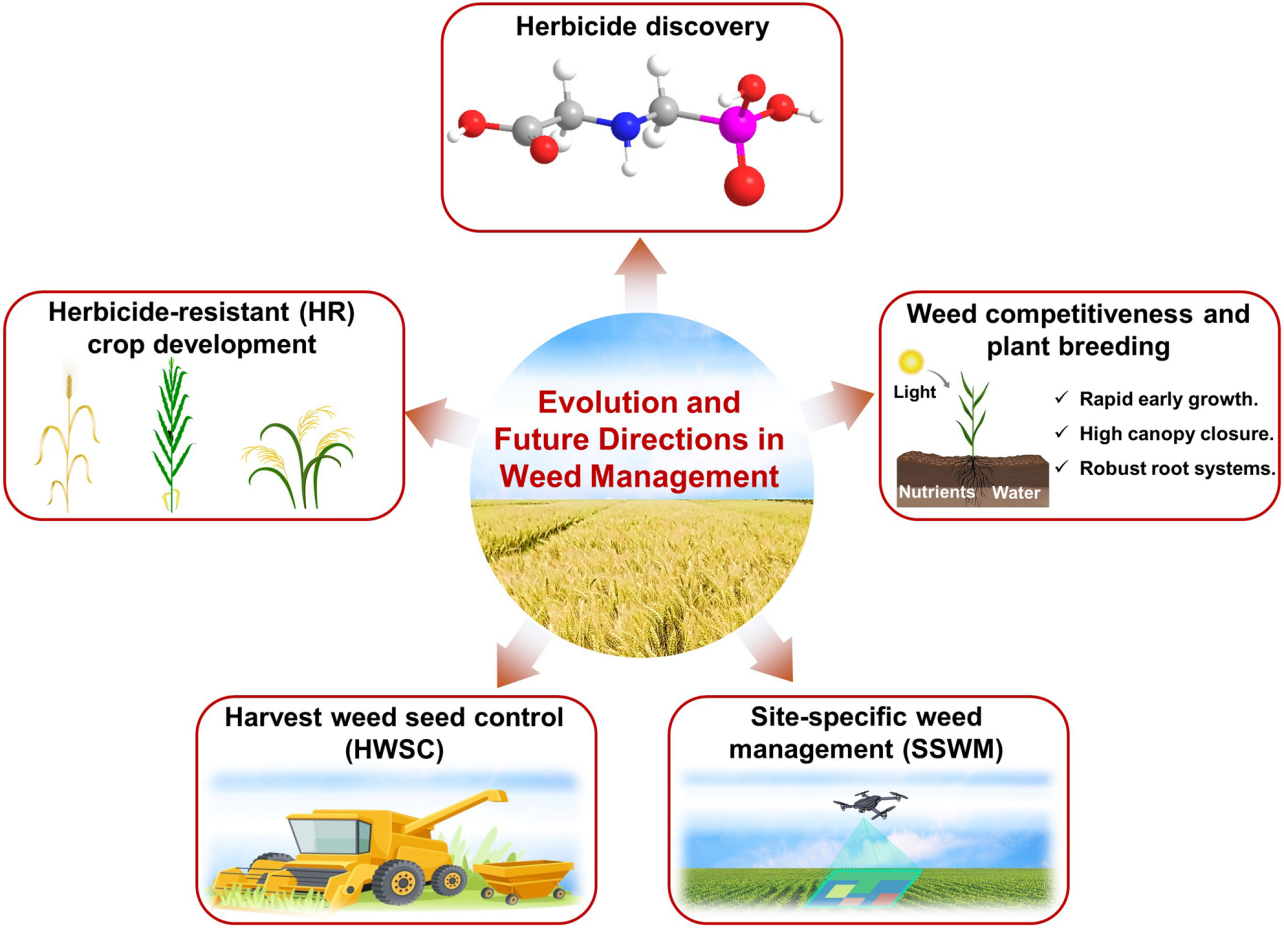
An overview of the evolution and future directions in weed management reveals a diverse array of strategies. Among these, herbicide discovery and the development of herbicide-resistant (HR) crops remain the most widely adopted approaches. In contrast, harvest weed seed control (HWSC) and site-specific weed management (SSWM) represent non-chemical strategies aimed at precision control. Additionally, enhancing crop competitiveness through plant breeding is categorized under biological control methods

The quest to enhance agricultural productivity is critically linked to effective weed management, given that weed infestation poses a significant threat to food security worldwide. As the global population continues to rise, which is projected to reach nearly 9.7 billion by 2050 (United Nations 2022), the demand for agricultural systems to increase yield and efficiency becomes even more immense. The severity of weed proliferation, which can drastically reduce crop yields and undermine food supplies, highlights the urgent need for innovative and sustainable management strategies (Horvath et al. 2023). This challenge has propelled the development of herbicide-resistant (HR) crops (Fig. 1), which are now a cornerstone in modern agronomy (Powles and Yu 2010; Clay 2021). These efforts reflect the vital necessity of developing effective weed management practices to sustain global food security amidst increasing environmental and economic pressures.

Herbicides have been pivotal in managing weeds, crucially supporting crop yields. However, the emergence of HR weeds presents a significant challenge, undermining the effectiveness of chemical weed control and potentially jeopardizing global food security (Heap 2014; Horvath et al. 2023). The development of HR crops represents a significant breakthrough. These crops are engineered to tolerate specific herbicides, allowing for the targeted elimination of competing weeds without harming the crops (Gaines et al. 2020). This advancement has revolutionized agricultural practices, facilitating more precise and efficient weed management strategies.

Despite their initial success, the widespread adoption of HR crops has inadvertently fostered the evolution of HR weed populations, presenting a complex challenge that emphasizes the need for a more holistic approach to weed management (Mendes et al. 2022). The ongoing “arms race” between the development of new herbicides and the rapid adaptation of weed populations necessitates a re-evaluation of existing strategies and an exploration of IWM practices. IWM advocates for a diversified approach, combining chemical, biological, mechanical, and cultural strategies to manage weed populations sustainably (Gonzalez-Andujar 2023) (Fig. 1). This paradigm shift towards a more integrated methodology highlights the industry’s recognition of the limitations of relying solely on chemical control. In parallel, advances in technology have ushered in an era of precision agriculture, offering innovative tools like site-specific weed management (SSWM) to enhance the efficiency and efficacy of weed control measures (Gerhards et al. 2022).

This review aims to navigate the intricate landscape of HR crop development and weed management. It will explore the historical trajectory, current state, and prospective future of herbicide resistance in crops and weeds, emphasizing the integration of traditional practices with cutting-edge technologies to forge a sustainable path forward in agricultural weed management. By dissecting the successes, challenges, and evolving strategies in this domain, the review offers insights and guidance for researchers, practitioners, and policymakers engaged in the ongoing quest to balance agricultural productivity with environmental stewardship.

## Herbicide Discovery

### Types of Herbicides and the Changing Landscape of Herbicide Development

**Fig. 2:**
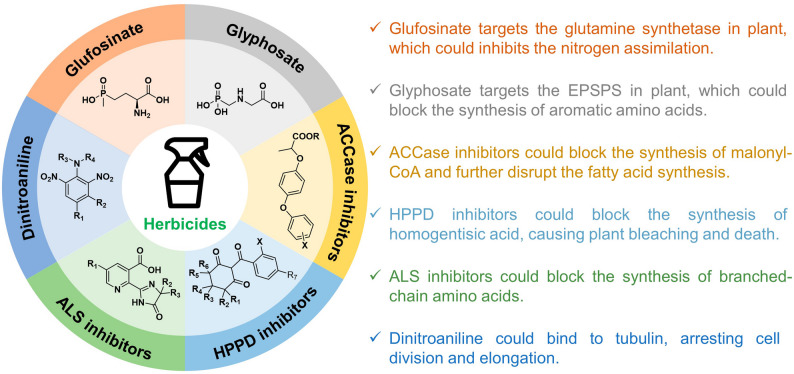
Overview of herbicide modes of action (MOAs) and target metabolic pathways. Several commonly used herbicides based on their modes of action and specific targets within plant metabolism are highlighted. Each section represents a different class of herbicides, emphasizing the chemical structure of typical compounds and their respective action sites. The key indicates how these herbicides disrupt essential physiological processes, for example, glutamine synthetase inhibition by glufosinate, EPSPS inhibition by glyphosate, and interference with pathways involved in nitrogen assimilation, amino acid synthesis, and cell division

The discovery and development of new herbicides have historically been pivotal in advancing agricultural productivity and weed management. The types of herbicides and their mechanisms of action are crucial in guiding the development of HR crops. Herbicides generally inhibit essential plant processes, and resistance can often be conferred through target site mutations or enhanced metabolic detoxification (Jugulam and Shyam 2019; Gaines et al. 2020). For instance, ALS (acetolactate synthase) inhibitors (Whitcomb 1999; Loubet et al. 2021) and ACCase (acetyl-CoA carboxylase) inhibitors (Takano et al. 2020b), which comprise a significant portion of the herbicide resistance market, typically block the synthesis of essential branched-chain amino acids in plants (Jin et al. 2022) (Fig. 2). Resistance to these herbicides is often conferred by inducing mutations at critical sites within this enzyme. Similarly, EPSPS (5-enolpyruvyl shikimate-3-phosphate synthase) inhibitors like glyphosate, a widely used non-selective herbicide, inhibit the shikimate pathway, which is crucial for the synthesis of aromatic amino acids (Alarcón-Reverte et al. 2015; Achary et al. 2020). Targeted mutations in the *EPSPS* gene can prevent glyphosate from binding to its target, thus conferring resistance. Currently, a variety of weeds have gained resistance to glyphosate through site mutations (Table 1).

**Table 1:** Representative *EPSPS* point mutations associated with glyphosate resistance across grass and broadleaf weeds

Amino acid substitution	Grass species and year first documented	Resistance level	References
Thr102Ser	*Tridax procumbens* (2018)	M	Li et al. (2018)
Pro106Ser	*Bidens pilosa* (2016)	H	Alcántara-de la Cruz et al. (2016)
	*Eleusine indica* (2002)	M	Baerson et al. (2002)
	*Lolium rigidum* (2008)	M	Simarmata and Penner (2008)
Pro106Thr	*E. indica* (2003)	M	Ng et al. (2003)
	*L. rigidum* (2006)	M	Wakelin and Preston (2006)
Pro106Ala	*L. rigidum* (2007)	M	Yu et al. (2007)
	*Lolium perenne ssp. multiflorum* (2008)	M	Jasieniuk et al. (2008)
Pro106Leu	*E. indica* (2015)	M	Chen et al. (2015)
	*L. rigidum* (2011)	M	(Kaundun et al. 2011)
Pro106Ser	*L. perenne ssp. multiflorum* (2007)	M	Perez-Jones et al. (2007)
	*Amaranthus tuberculatus* (2013)	M	Nandula et al. (2013)
	*Echinochloa colona* (2013)	M	Alarcón-Reverte et al. (2013)
	*L. perenne* (2015)	H	Ghanizadeh et al. (2015)
Thr102Ile/Pro106Ser	*Bidens pilosa* (2016)	H	Alcántara-de la Cruz et al. (2016)
	*E. indica* (2015)	H	Yu et al. (2015), Chen et al. (2015)
Thr102Ile/Pro106Thr	*Bidens subalternans* (2019)	H	Takano et al. (2020a)
Thr102Ile/Ala103Val/Pro106Thr	*Amaranthus hybridus* (2019)	H	Perotti et al. (2019)

Amino acid positions are numbered according to the *Arabidopsis thaliana EPSPS* sequence. In the resistance-level column, M indicates moderate resistance (< 10-fold relative to the sensitive biotype), whereas H indicates high resistance (> 10-fold relative to the sensitive biotype).

Although these discoveries revolutionized weed management, their widespread and repeated use over successive cropping cycles has inadvertently intensified selection pressure on weed populations, thereby accelerating the evolution of herbicide resistance. The emergence of herbicide resistance in weeds is driven by complementary genetic and physiological mechanisms. Target-site resistance typically originates from spontaneous mutations in genes encoding herbicide target enzymes such as ALS, ACCase, and EPSPS (Heap 2014). Although these mutations usually occur at very low frequencies within the soil seed bank, sustained herbicide pressure selectively enriches resistant individuals, allowing them to survive, reproduce, and progressively dominate the population. In contrast, non-target-site resistance generally arises from physiological adaptations such as enhanced metabolic detoxification, reduced herbicide translocation, or compartmentalization, which enable weeds to tolerate herbicide exposure without alterations at the molecular target site (Jugulam and Shyam 2019).

In natural weed populations, most resistance evolution begins with a few individuals carrying spontaneous target-site mutations that pre-exist in the seed bank. When herbicides with the same mode of action (MOA) are applied repeatedly, these resistant genotypes are preferentially selected, and their seeds replenish the seed bank, amplifying resistant alleles across generations. Physiological or metabolic resistance can also accumulate over time through the soil seed bank, highlighting the importance of diversified management and early resistance surveillance (Heap 2014; Peterson et al. 2018; Baucom 2019).

Without diligent monitoring, crop rotation, and herbicide diversification, resistant genotypes may proliferate, ultimately dominating the population and replenishing the soil seed bank with resistant progeny. However, the pace of introducing new MOAs has significantly slowed since the late 20th century, with no new MOA commercialized in over three decades (Dayan 2019; He et al. 2022). Currently, the most widely used herbicides include glyphosate, glufosinate, ALS inhibitors, ACCase inhibitors, HPPD (hydroxyphenylpyruvate dioxygenase) inhibitors, and dinitroanilines (Fig. 2). This slowdown coincides with the widespread adoption of glyphosate-resistant crops, which fostered a predominant reliance on glyphosate and reduced the industry’s incentive to develop novel herbicides (Baek et al. 2021; Metcalfe et al. 2024). The resurgence of HR weed populations therefore highlights the urgent need for innovative herbicidal solutions. Despite challenges such as escalating development costs estimated at approximately $300 million per new product and the decade-long timeline from discovery to commercialization, the agrichemical industry has reignited its efforts in herbicide discovery (Beckie et al. 2019; Sleugh et al. 2021).

### Innovation in Herbicide Discovery Through Natural Metabolites and Advanced Screening

Recent trends in herbicide discovery have increasingly focused on exploring natural products, particularly plant and microbial metabolites, as potential sources for new herbicidal compounds. Research indicates that natural phytotoxins may unveil novel target sites for herbicidal action (Dayan and Duke 2014; Berestetskiy 2023). For instance, pyrenophorol, a macrodiolide isolated from a *Drechslera avenae* pathotype, has been suggested as a selective herbicide for *Avena fatua* (L). Metabolic fingerprinting has revealed that pyrenophorol utilizes a unique MOA distinct from the existing herbicide MOAs (Kastanias and Chrysayi-Tokousbalides 2000; Aliferis and Chrysayi-Tokousbalides 2006). Additionally, the study of allelochemicals, substances plants produce to suppress the growth of competing species, has also highlighted potential new herbicidal MOAs (Li et al. 2021; Valiño et al. 2023). Notably, water-soluble extracts from *Artemisia argyi* have been shown to inhibit the germination and growth of weeds through multiple targets and pathways, demonstrating their potential for herbicide development (Li et al. 2021). Integrating genomics, metabolomics, and computational modelling have revolutionized the herbicide discovery process. High-throughput screening techniques now facilitate the rapid evaluation of thousands of compounds for herbicidal activity (Dayan 2019), while molecular modelling and structure-activity relationship studies aid in designing molecules that specifically target plant enzymes or pathways, potentially leading to the discovery of herbicides with novel MOAs (He et al. 2022; Leng et al. 2023; Li et al. 2023).

### The Future of Herbicide Discovery Amidst Growing Resistance and Regulatory Complexities

Despite these advancements, discovering new herbicides remains fraught with challenges. The evolution of herbicide resistance, regulatory hurdles, environmental concerns, and the economic risks associated with developing new herbicides are significant obstacles (Peterson et al. 2018; Baucom 2019; Mendes et al. 2022). However, the growing crisis of HR weeds continues to drive the demand for innovative solutions, presenting opportunities for breakthroughs in the field. The future of herbicide discovery likely lies in a multi-faceted approach that combines traditional chemistry with cutting-edge technology and a deeper understanding of plant biology. Collaborative efforts between academia, industry, and regulatory bodies will be crucial in fostering an environment conducive to innovation while ensuring the safety and sustainability of new herbicidal products (Espig et al. 2023; Dwivedi et al. 2024). As the agronomic landscape evolves, the continuous pursuit of novel herbicides will be vital in developing integrated weed management strategies to sustain agricultural productivity and environmental health in the face of mounting challenges.

## Development of Herbicide-Resistant Crops

### The Development of Herbicide-Resistant Crops and Continuous Evolvement of Enabling Genetic Modification Technologies

The advent of HR crops is fundamentally dependent on sophisticated genetic engineering techniques, representing a significant milestone in agricultural biotechnology. The primary mechanism for rendering crops resistant to herbicides involves introducing genes that enable plants to degrade or tolerate the herbicide (Olchanheski et al. 2014; Jin et al. 2022). These crops have been genetically modified to withstand specific herbicides, allowing farmers to apply these chemicals without damaging the crop, thereby providing an efficient means to control a broad spectrum of weeds. For instance, glyphosate-resistant crops possess a version of the *EPSPS* gene that is not inhibited by glyphosate, allowing the plants to survive its application (Achary et al. 2020; Ouyang et al. 2021). Similarly, crops resistant to glufosinate contain a gene that detoxifies the herbicide within the plant (Green and Owen 2011; Mall et al. 2019).

Traditional gene modification methods like *Agrobacterium*-mediated transformation and biolistic particle delivery systems have historically been utilized to introduce foreign DNA into plant genomes, conferring HR traits (Chen et al. 2022). The first HR crops were introduced in the mid-1990s, with the development of glyphosate-resistant soybeans marking a revolutionary step in crop production (Padgette et al. 1995). The adoption of HR crops has grown exponentially since then, with key crops including soybean, maize, cotton, and canola being engineered for resistance to various herbicides (Brookes and Barfoot 2015; Beckie et al. 2019). These methods have enabled the integration of genes such as *phosphinothricin acetyltransferase* (*PAT*), which imparts resistance to glufosinate (Cui et al. 2016).

Recent advancements have seen a shift towards more precise genetic engineering technologies, notably CRISPR/Cas9 (Hwarari et al. 2024). This revolutionary technique allows for exact genetic alterations by targeting specific DNA sequences within the plant genome, which can introduce or repair mutations without incorporating foreign DNA. CRISPR/Cas9 has been instrumental in developing resistance to various herbicides by editing genes directly involved in their biochemical mechanisms (Dong et al. 2021; Hussain et al. 2021; Yang et al. 2022). Moreover, the CRISPR/Cas system has evolved to include more refined technologies like base editing and prime editing (Kantor et al. 2020). These advancements enable the introduction of precise point mutations without the associated risks of double-stranded DNA breaks, which are typical of traditional CRISPR/Cas9 applications. Base editing, for instance, can directly convert one DNA base into another, subtly altering the genetic code at specific loci to confer herbicide resistance (Wang et al. 2024). This method has been applied to modify the *ALS*,* ACCase*, and *EPSPS* genes, thereby enhancing crop tolerance to various herbicides with remarkable specificity and minimal off-target effects. For example, modifying the *ALS* and *ACCase* genes through base editing was shown to produce HR wheat lines that confer tolerance to multiple herbicide types, including sulfonylurea, imidazolinone, and aryloxyphenoxy propionate (Zhang et al. 2019).

### The Dynamics of Stacked Herbicide-Resistant Traits and Their Broader Implications in Agriculture

In the ongoing battle against HR weeds, agricultural biotechnology has progressed from developing crops resistant to a single MOA to creating varieties that carry multiple HR traits. This strategy, known as trait stacking, is essential for effective weed management in modern agriculture. Trait stacking involves integrating multiple resistance genes into a single crop variety, allowing the use of different herbicides that act on various biological pathways within the weeds. This not only broadens the spectrum of controlled weed species but also helps in delaying the development of resistance by reducing the selective pressure exerted by any single herbicide (D’Halluin et al. 2013; Vennapusa et al. 2022).

For example, developing soybean varieties resistant to glyphosate and dicamba represents a significant advancement in HR crop technology (Byker et al. 2018). Glyphosate inhibits the EPSPS enzyme crucial for aromatic amino acid synthesis, while dicamba mimics natural plant hormones, causing uncontrolled growth and leading to the death of susceptible plants (Leino et al. 2021). By stacking these traits, farmers can target a wider range of weed species with different herbicides, effectively managing those that have developed resistance to glyphosate alone (Chopra et al. 2020). Another notable example is the development of maize varieties that combine resistance to both atrazine (a photosynthesis inhibitor) and 2,4-D (a synthetic auxin herbicide). This combination is particularly effective against broadleaf and grass weeds, providing a comprehensive control strategy that mitigates the risk of developing resistance to either herbicide when used in isolation (Moinuddin et al. 2018; Nandula 2019). Advances in genetic engineering, particularly gene editing technologies like CRISPR/Cas9, have made it feasible to precisely insert multiple herbicide-resistance genes into crop genomes without the linkage drag associated with traditional breeding methods (Hussain et al. 2021). These innovations ensure that crops can withstand various herbicidal actions, thus preserving the effectiveness of herbicides and sustaining agricultural productivity.

### The Future of Herbicide-Resistant Crops in Sustainable Weed Management

**Fig. 3:**
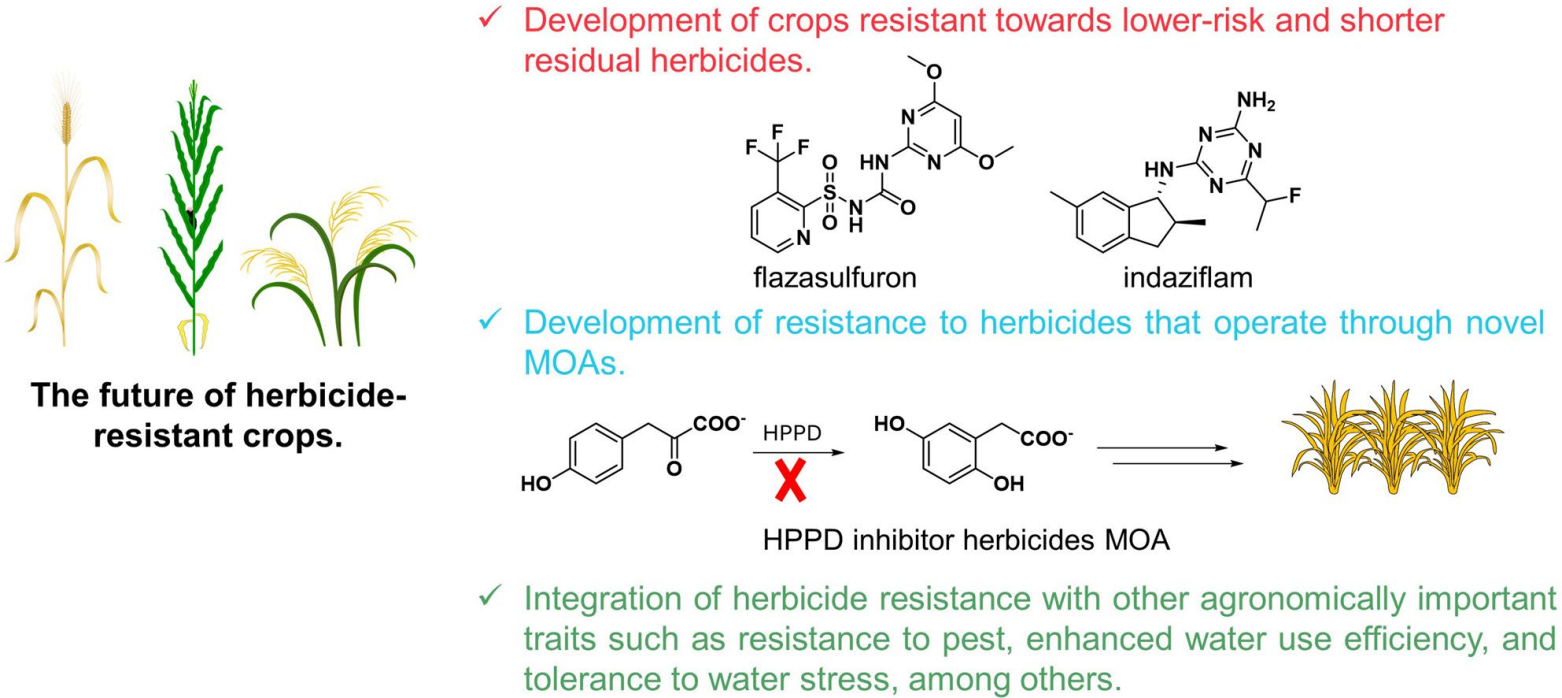
Future directions of herbicide-resistant (HR) crops in sustainable weed management. This illustration depicts prospective advancements in HR crop development with an emphasis on sustainability. The schematic highlights cereal crops alongside emerging herbicide technologies, illustrating the shift towards lower-risk and shorter-residual herbicides, such as flazasulfuron and indaziflam, which function through novel modes of action (MOAs). The figure also outlines the biochemical pathway of hydroxyphenylpyruvate dioxygenase (HPPD)-inhibiting herbicides and shows how these innovations can be integrated with complementary agronomic traits, including pest resistance, enhanced water use efficiency, and tolerance to abiotic stress, to promote agricultural sustainability

As the agricultural sector adapts to the challenges of sustainability and environmental stewardship, the focus on developing HR crops is shifting towards traits that confer resistance to newer and more environmentally benign herbicides. This shift is crucial as it aligns with global efforts to reduce the ecological footprint of agriculture while maintaining the efficacy of weed management strategies (Hasan et al. 2021). One example of this shift is the development of crops resistant to herbicides that have a lower risk of leaching into waterways and a shorter residual presence in the environment (Fig. 3). Herbicides like flazasulfuron and indaziflam, noted for their low application rates and minimal non-target toxicity, are becoming more popular in HR crop development (Sebastian et al. 2017; Soares et al. 2023). By engineering crops that can tolerate these herbicides, farmers can use them more effectively without the risk of harming nearby ecosystems or non-target plant species.

Another area of focus is the development of resistance to herbicides that operate through novel MOAs (Fig. 3). This is particularly important as resistance to traditional herbicides like glyphosate continues to grow. For instance, researchers are exploring the integration of resistance to HPPD inhibitors, which are crucial for controlling a broad spectrum of weed species while being less harmful to the environment (Lin et al. 2023). Crops engineered to resist such herbicides benefit from their efficiency and the reduced likelihood of developing cross-resistance with older types of herbicides.

Furthermore, integrating herbicide resistance with other agronomically important traits, such as insect resistance, is becoming increasingly vital in modern agriculture (Fig. 3). This trait-stacking strategy enhances the crop’s ability to withstand multiple stress factors, ensuring productivity under varied environmental conditions. A notable example is Syngenta’s maize Agrisure^®^ event Bt11. This particular genetic modification incorporates a *PAT* gene expression cassette, which provides tolerance to glufosinate, and a *Cry1Ab* gene expression cassette, which offers resistance to the European corn borer, a common pest affecting maize crops. This dual-trait stacking enhances crop resilience and productivity by protecting against specific pests and facilitating easier weed management. Similarly, soybean varieties have been developed that combine resistance to glyphosate with enhanced water use efficiency (Zobiole et al. 2010). These varieties allow farmers to use glyphosate to control weeds without fear of damaging the crop, while the improved water efficiency trait ensures that the plants can better manage stress during periods of low water availability. This dual trait significantly improves yield stability and resource use efficiency, which are key factors for sustainable farming practices (Ingrao et al. 2023). Furthermore, cotton has seen advancements where varieties have been engineered to include both herbicide resistance and traits that confer tolerance to high temperatures and water stress (Vulchi et al. 2022). These varieties ensure that cotton cultivation can remain viable in regions experiencing hotter and drier growing seasons, thereby securing the livelihoods of farmers and the cotton industry’s sustainability.

By stacking these traits, breeders not only address the specific needs of diverse agro-ecological zones but also contribute to the resilience of agricultural systems against the backdrop of global climate change. These developments highlight the importance of multi-trait genetic engineering as a viable approach to enhance both the environmental sustainability and economic viability of global agriculture.

The widespread adoption of HR crops has significantly impacted agricultural practices, with implications for economic sustainability and environmental health. While HR crops have simplified weed control and potentially increased yields, they have also led to a dependency on chemical control measures, raising concerns about biodiversity, soil health, and herbicide runoff (Schütte et al. 2017; Yang et al. 2024).

Moreover, there is a growing emphasis on integrating HR crops within a holistic weed management framework that incorporates cultural, mechanical, and biological control methods to ensure sustainable crop production and mitigate the risk of resistance development (Riemens et al. 2022; Gonzalez-Andujar 2023). The development and deployment of HR crops are subject to regulatory approvals, which assess potential environmental and health impacts. Public acceptance varies globally, influenced by perceptions of safety, environmental concerns, and the socio-economic impacts of adopting genetically modified organisms (GMOs) in agriculture (Naveen and Sontakke 2024).

## Preemergence Herbicides

### The Renewed Role of Preemergence Herbicides in Managing Herbicide-Resistant Weeds

Escalating challenges posed by HR weeds have catalyzed a strategic shift in weed management practices, with a growing emphasis on the use of preemergence (PRE) herbicides (Metcalfe et al. 2018; Striegel et al. 2021). These herbicides, applied to the soil before the weeds germinate, have garnered attention for their role in circumventing the limitations of postemergence (POST) herbicide applications, particularly in scenarios where weed populations have developed resistance (Mitra et al. 2022).

PRE herbicides are essential for managing weeds before they emerge by forming a barrier in the soil that impedes weed germination and growth. Examples include pendimethalin, used in wheat, corn, and soybeans to inhibit cell division (Ramasahayam 2014); trifluralin, applied in cotton and vegetables to block root development (Grover et al. 1997); S-metolachlor for controlling annual grasses in corn and soybeans by affecting shoot elongation (O’Connell et al. 1998); isoxaben, which inhibits cellulose synthesis in ornamentals (Desprez et al. 2002); and prodiamine, used in turf to prevent weed seed germination (Zulfiqar 2021) (Table 2). Additionally, atrazine serves dual purposes in corn fields to inhibit photosynthesis in both PRE and POST applications (Zhu et al. 2009) (Table 2). These herbicides are selected based on crop type, target weeds, and environmental factors, requiring careful timing and application to maximize effectiveness and minimize environmental risks (Senseman 2007). The agronomic importance of these herbicides transcends their direct weed control benefits; they also facilitate the optimization of crop competitiveness. By suppressing early weed competition, PRE herbicides ensure that crops can establish dominance, thereby improving resource utilization (light, nutrients, and water) and ultimately enhancing yield potential (Sen et al. 2021).

**Table 2:** Chemical structures and MOAs of several typical PRE herbicides

Pre-emergent herbicides	Chemical structure	Mode of action	References
Pendimethalin		Inhibits cell division	Ramasahayam (2014)
Trifluralin		Blocks root development	Grover et al. (1997)
S-metolachlor		Affects shoot elongation	O’Connell et al. (1998)
Isoxaben		Inhibits cellulose synthesis	Desprez et al. (2002)
Prodiamine		Prevents weed seed germination	Zulfiqar (2021)
Atrazine		Inhibits photosynthesis	Zhu et al. (2009)

### The Impact of Soil-Residual Activity of Preemergence Herbicides on Long-Term Weed Management

A critical feature of PRE herbicides is their soil-residual activity, which provides a window of weed control extending beyond the initial application (Silva et al. 2023). This sustained activity is essential for combating weeds that germinate over a prolonged period, thereby protecting crops from late-emerging weed populations that could compete for resources. For example, herbicides like pendimethalin and prodiamine are known for their residual action in the soil, effectively controlling weed emergence throughout the growing season in crops such as soybeans and ornamental plants (Altland 2019).

The effectiveness of these soil-residual herbicides, however, can vary significantly based on several soil and environmental factors. Soil type, for instance, affects the herbicide’s persistence and bioavailability (Takeshita et al. 2019). Sandy soils with low organic matter may not retain the herbicide as effectively as clay soils, reducing the duration of weed control. Conversely, the organic matter content of the soil can influence the binding of the herbicide, with higher organic matter potentially leading to reduced efficacy due to increased adsorption. Environmental conditions such as temperature and rainfall also play critical roles (Varanasi et al. 2016). For instance, excessive rainfall can lead to herbicide leaching, particularly in sandy soils, which diminishes the herbicidal barrier and may require reapplication to maintain effective weed control. Conversely, drought conditions can reduce the herbicide’s effectiveness by limiting its dispersion in the soil.

Given these variables, the selection and application of soil-residual herbicides necessitate a thorough understanding of local agronomic and environmental conditions to optimize their efficacy. Adjusting application rates and timing according to soil characteristics and weather patterns can greatly enhance the performance of PRE herbicides, ensuring continuous protection against weed infestations and supporting sustainable crop production (Silva et al. 2023; Vagedes et al. 2024).

### Enhancing Weed Management with Preemergence Herbicides in an Integrated Pest Management Framework

Incorporating PRE herbicides into an integrated IWM framework enhances the diversity of weed control tactics, a fundamental principle for delaying the evolution of herbicide resistance. When used in conjunction with other control methods—mechanical, cultural, and biological—PRE herbicides contribute to a more robust and sustainable weed management strategy (Mitra et al. 2022).

While PRE herbicides offer numerous benefits, their deployment must be judiciously managed to avoid off-target effects and minimize environmental impact. The potential for leaching, runoff, and impact on non-target species mandates a careful balance between effective weed control and environmental stewardship (Vagedes et al. 2024). Moreover, selecting PRE herbicides must account for their spectrum of activity, persistence in the soil, and potential interactions with subsequent crops in the rotation (Sharma et al. 2021; Varah et al. 2024). As the agricultural sector continues to evolve, the role of PRE herbicides will likely expand, particularly in systems seeking to reduce reliance on POST applications due to resistance issues. Ongoing research is essential to develop new active ingredients with novel MOAs and to refine application techniques that maximize efficacy while minimizing environmental risks.

## Weed Competitiveness and Plant Breeding

### Enhancing Crop Competitiveness to Naturally Suppress Weeds

The evolution of HR weeds has necessitated the exploration of alternative weed management strategies, one of which is enhancing the weed-competitive ability of crops through plant breeding (Kaur et al. 2021). This approach aims to develop cultivars that can effectively suppress weeds through their inherent growth characteristics, reducing reliance on herbicides and contributing to sustainable agriculture practices.

Weed-competitive crop cultivars are designed to effectively compete with weeds for resources such as light, nutrients, and water. These cultivars exhibit traits like rapid early growth, high canopy closure, and robust root systems, which enable them to outcompete neighbouring weeds, thereby reducing the weed biomass and seed bank in the soil (Kucek et al. 2021). Plant breeders have started to incorporate weed-competitive traits as selection criteria in breeding programs. This shift is driven by the understanding that crops with aggressive early growth can shade out weeds, reducing their ability to photosynthesize and survive. Traits such as taller stature, greater leaf area, and faster canopy closure are among those selected to enhance the competitive ability of crop cultivars against weeds (Xu et al. 2023).

### The Role of Weed-Competitive Cultivars in Enhancing Yield Stability and Sustainable Farming

Apart from reducing weed pressure, weed-competitive cultivars can contribute to yield stability and sustainability in agricultural systems. By suppressing weed growth, these cultivars can minimize the need for herbicide applications, thereby reducing the selection pressure for herbicide resistance (Weiner 2023). Furthermore, this strategy aligns with IWM principles, offering a holistic approach to weed control (Andrew et al. 2015). While the concept is promising, breeding for weed competitiveness poses several challenges. Weed-competitive traits need to be effectively integrated without compromising other desirable agronomic characteristics, such as yield and disease resistance. Moreover, the effectiveness of weed-competitive cultivars can vary based on environmental conditions and the weed species present, necessitating region-specific breeding efforts (Weiner 2023). Research has demonstrated the potential of weed-competitive cultivars in various crops. For example, studies in wheat have shown that cultivars with rapid early growth and higher biomass can significantly reduce the abundance of weeds like ryegrass (*Lolium rigidum* Gaud) and fumitory (*Fumaria* spp. L.) (Kaur et al. 2021; Hendriks et al. 2022). Similarly, in rice, cultivars with specific growth habits have been effective in suppressing weed growth, contributing to higher yield and reduced herbicide dependence (Dass et al. 2017).

### Leveraging Genomic Tools To Develop Weed-Competitive Cultivars for Sustainable Weed Management

Integrating modern genomic tools and phenotyping technologies can accelerate the development of weed-competitive cultivars. Collaborative efforts between weed scientists and plant breeders are essential to ensure that the development of weed-competitive cultivars aligns with the overall goals of sustainable weed management (Bagavathiannan et al. 2019). Genomic tools such as marker-assisted selection (MAS), genome-wide association studies (GWAS), and gene editing technologies like CRISPR/Cas9 are pivotal in this process. These tools enable breeders to pinpoint genetic loci associated with traits that improve a plant’s competitiveness, such as rapid early growth, greater root biomass, and allelopathic capabilities. For example, traits that enable faster and denser canopy development can shade out weeds, effectively suppressing their growth by limiting their access to sunlight (Huber et al. 2021). Researchers have successfully used MAS to develop rice varieties with enhanced early vigor and taller stature, traits that confer competitive advantages against weeds such as barnyard grass (Anandan et al. 2022). Similarly, GWAS has been instrumental in identifying allelopathic traits in wheat that deter the growth of surrounding weeds by releasing natural herbicidal compounds from the roots (Kaur et al. 2021). Furthermore, CRISPR/Cas9 technology offers the potential to directly edit genes responsible for competitive traits, providing a precise method to enhance crop competitiveness without the lengthy process of traditional breeding. For instance, editing genes to improve root architecture could enable crops to more effectively compete for water and nutrients, an advantage in nutrient-limited soils (Rahim et al. 2024). This genomic-based strategy represents a forward-thinking approach to weed management, positioning weed-competitive cultivars as integral components of future farming systems.

## Harvest Weed Seed Control

### Harvest Weed Seed Control as a Pivotal Strategy for Reducing Herbicide Reliance

Harvest Weed Seed Control (HWSC) is an innovative approach that targets weed seeds during harvest to reduce future infestations and diminish reliance on herbicides. This strategy is particularly effective against weeds that are resistant to herbicides, as it physically removes or destroys their seeds before they can contribute to the soil seed bank (Akhter et al. 2023). Techniques within HWSC include chaff lining, where chaff and seeds are funneled into narrow rows at harvest, concentrating weed seeds in specific areas for easier management. Chaff tramlining involves directing the chaff into permanent wheel tracks where subsequent traffic crushes the seeds, preventing germination.

One of the most notable HWSC technologies is the Harrington Seed Destructor, which integrates with harvesting equipment to crush weed seeds as crops are processed. This device has proven effective in drastically reducing the number of viable weed seeds returned to the field, thereby interrupting the reproduction cycle of HR weed species (Jacobs and Kingwell 2016). For instance, studies have shown that the Harrington Seed Destructor can destroy up to 99% of targeted weed seeds, significantly reducing subsequent weed emergence (Schwartz-Lazaro et al. 2017).

However, the success of HWSC largely depends on the degree of weed seed retention at the time of crop harvest (Akhter et al. 2023). For instance, cleavers (*Galium* spp.) and wild mustard (*Sinapis arvensis* L.) retain approximately 74% and 70% of their seeds, respectively, making them excellent potential targets for HWSC implementation. In contrast, blackgrass (*Alopecurus myosuroides*) exhibits a high level of seed shattering before harvest, which may limit the effectiveness of HWSC systems against this species (Walsh et al. 2018). Therefore, the decision to adopt HWSC should be based on the seed retention characteristics of dominant weed species within a given cropping system to ensure effective and practical weed management outcomes.

### Optimizing Harvest Weed Seed Control for Effective Weed Management

Effective HWSC involves timing, technology selection, and integration with other weed management strategies to maximize its impact on reducing the weed seed bank at harvest (Akhter et al. 2023). Timing is crucial in HWSC; the process should be aligned with the peak maturity of weed seeds but before they shed to the ground. This requires careful monitoring of weed growth stages throughout the cropping season to determine the optimal harvest time. Delayed harvesting can lead to significant seed shedding, reducing the efficacy of HWSC techniques (Walsh and Powles 2022). The choice of HWSC technology is also vital. Tools like the Harrington Seed Destructor, chaff liners, and chaff decks are designed to target and destroy weed seeds during the harvest. Each tool has its own specific application and effectiveness, depending on the crop type and the predominant weed species (Jacobs and Kingwell 2016). For example, the Harrington Seed Destructor has been shown to be extremely effective in destroying seeds from rigid ryegrass and wild radish, common in many grain crops (Walsh and Powles 2022). Integrating HWSC with other management practices, such as crop rotation, strategic tillage, and chemical control, can also enhance its effectiveness. Crop rotation can suppress specific weed species, strategic tillage can disrupt weed seed germination, and judicious use of herbicides can control grown weeds, reducing the seed input into the HWSC system (Walsh 2018). Furthermore, ongoing research and development are essential to improving the efficiency of HWSC equipment and techniques. Innovations that reduce cost, increase ease of use, and enhance seed destruction rates will help make HWSC a more accessible and effective option for farmers worldwide, supporting sustainable agricultural practices (Somerville et al. 2018).

## Site-Specific Weed Management

### Site-Specific Weed Management for Enhanced Efficiency and Sustainability in Agriculture

SSWM represents a paradigm shift in agricultural weed control, transitioning from uniform field treatment to more targeted, precise interventions (Lati et al. 2021). By identifying and addressing weed infestations at a granular level, SSWM optimizes herbicide usage, enhances weed control efficacy, and mitigates the environmental impact associated with over-application of chemicals. SSWM utilizes advanced technologies such as GPS mapping, drones, and sensor-based systems to accurately identify weed densities and locations within a field. This spatial awareness allows for precise herbicide applications, targeting only the areas that require treatment and thereby conserving herbicides, reducing costs, and minimizing environmental contamination (Esposito et al. 2021).

Precision agriculture has revolutionized SSWM by enabling more targeted and efficient weed control strategies that conserve resources and enhance crop productivity. High-resolution satellite imagery, drones equipped with advanced sensors, and ground-based robotic systems are pivotal in mapping weed infestations with high accuracy (Sapkota et al. 2023). Variable rate technology (VRT) further refines this process by adjusting herbicide application rates in real time according to weed density and species composition (Sökefeld 2010). This precision prevents over-application and helps manage herbicide resistance by reducing unnecessary chemical exposure to weed populations. Additionally, integrating machine learning algorithms and artificial intelligence (AI) further refines data analysis, enabling real-time decision-making and operational adjustments (Rai et al. 2023). AI algorithms can predict weed emergence and growth patterns based on historical data and current field conditions, allowing for pre-emptive management actions that are more effective and less reliant on herbicides (Vijayakumar et al. 2023). The advent of autonomous robots equipped with advanced vision systems and mechanical tools offers the potential for mechanical weed removal or spot-spraying of herbicides. These robots can navigate fields independently, identify weeds, and perform precise actions, such as pulling or cutting weeds or applying herbicides directly to target areas, further reducing the need for broad-spectrum chemical applications (Baltazar et al. 2021).

Moreover, SSWM can be integrated with other management practices, such as cover cropping and mechanical weeding. Cover crops can suppress weed growth by competing for resources, while mechanical weeding can remove established weeds without chemical intervention. For instance, incorporating a cover crop like cereal rye can effectively suppress winter weeds, reducing the need for early-season herbicide applications (Blank et al. 2023).

### Overcoming Barriers To the Adoption of Site-Specific Weed Management

Despite its potential, the widespread adoption of SSWM faces several challenges. One of the primary obstacles to adopting SSWM is the high initial cost and complexity of the technology required. Precision agriculture tools, which are fundamental for SSWM, often come with a steep price tag and require significant technical expertise to operate effectively. This can be particularly prohibitive for small- to medium-sized enterprises that may not have the capital or the technical staff to implement these technologies. Researchers like Monteiro and Santos (2022) emphasize the need for cost-effective technology solutions and training programmes that can mitigate these challenges (Monteiro and Santos 2022). Another significant barrier is the variability in effectiveness, which can be attributed to the diverse nature of fields and weed populations. Satterfield et al. (2017) noted that SSWM systems must be highly adaptable to local conditions to be truly effective (Satterfield et al. 2017). This requires detailed, site-specific data, which can be tedious and expensive to collect. Moreover, the success of SSWM depends heavily on the accuracy of weed detection and mapping, which can be compromised by factors such as lighting conditions and plant overlap (Lati et al. 2021). Furthermore, there is a notable gap in regulatory support for SSWM. Regulatory frameworks often do not differentiate between traditional weed management techniques and precision approaches, which can discourage innovation and adoption. This is pointed out by Korres (2018), who argues for policies that specifically encourage precision weed management through incentives or subsidies (Korres 2018).

Despite these challenges, the potential benefits of SSWM, such as reduced environmental impact and increased cost efficiency in the long run, present a compelling case for its adoption. For SSWM to become more accessible, concerted efforts are necessary from technology developers, policymakers, and the agricultural community to address these barriers (Da Costa Lima and Mendes 2020).

### The Economic and Environmental Benefits of Site-Specific Weed Management

SSWM offers substantial economic and environmental benefits, making it a compelling approach for modern agriculture. Economically, SSWM can significantly reduce herbicide costs. By targeting only the areas where weeds are present, farmers can decrease the volume of herbicides used, leading to substantial cost savings (Sapkota et al. 2023). Research by Christensen et al. (2009) demonstrated that SSWM could reduce herbicide usage by up to 50%, directly impacting the bottom line for farmers through reduced input costs (Christensen et al. 2009).

The environmental benefits of SSWM are equally significant. Reduced herbicide use minimizes soil and water contamination, thereby preserving biodiversity and protecting aquatic ecosystems from harmful chemical runoff. This aspect of SSWM aligns well with sustainable farming practices and environmental conservation efforts. Furthermore, by maintaining a healthier soil biota, SSWM supports the soil’s natural fertility and its ability to sequester carbon, contributing to climate change mitigation. Moreover, SSWM contributes to more efficient land use. As detailed by Gerhards et al. (2022), precision weed control can improve crop yields by ensuring that crops face less competition from weeds for nutrients, sunlight, and space (Gerhards et al. 2022). This optimization of resource allocation not only boosts productivity but also enhances the overall sustainability of farming operations.

While technological and genetic innovations have significantly expanded the weed management toolbox, the long-term success of these strategies ultimately depends on proactive stewardship. Effective stewardship ensures that chemical, biological, and mechanical control tactics are implemented in a coordinated manner to delay herbicide resistance evolution and preserve the efficacy of existing weed management tools.

## Stewardship Guidelines for Delaying Herbicide Resistance Evolution in Weeds

Effective stewardship is fundamental to slowing the evolution of HR weed populations and preserving the efficacy of existing herbicides. Sustainable weed management relies on the responsible use of herbicides within a comprehensive framework that integrates agronomic, chemical, and ecological principles. The goal of stewardship is to extend herbicide longevity by minimizing selection pressure on weed populations and preventing the accumulation of resistant alleles within the soil seed bank. A proactive stewardship framework encompasses several key practices that operate synergistically to mitigate resistance development. Rotating or combining herbicides with MOAs across seasons diversifies selection pressure and slows the fixation of resistance alleles, while mixtures with complementary MOAs reduce the likelihood that a single mutation can confer cross-resistance. Embedding herbicide use within a broader IWM framework further enhances sustainability by incorporating complementary approaches such as crop rotation, cover cropping, mechanical weeding, and the use of weed-competitive cultivars to suppress weeds through ecological and agronomic means. Stewardship also emphasizes managing the soil seed bank, which serves as a long-term reservoir for resistant genotypes; practices such as HWSC, timely weed removal, and prevention of seed return to soil are critical for limiting the persistence and spread of resistance genotypes. In addition, advances in SSWM, variable-rate technologies, and precision spraying now allow localized herbicide applications based on spatial weed maps, thereby reducing herbicide inputs and selection pressure. Regular field scouting plays a complementary role by enabling early detection of resistant weed patches and facilitating rapid, targeted intervention. Equally important are education, monitoring, and policy support, as successful stewardship depends on coordinated action among farmers, extension agents, researchers, and policy makers. Continuous education, region-wide resistance surveillance programs, and industry-supported stewardship initiatives are essential to ensure compliance with best practices and promote early resistance detection and mitigation (Beckie et al. 2019; Espig et al. 2023; Varah et al. 2024). Collectively, these integrated stewardship measures constitute a proactive, evidence-based strategy for sustaining herbicide efficacy, protecting agroecosystem health, and maintaining agricultural resilience. Integrating stewardship practices into policy frameworks and farm-level decision-making is therefore vital for ensuring the long-term productivity and ecological sustainability of modern cropping systems.

## Conclusions and Future Outlook

The development of HR crops and the corresponding advancements in IWM strategies represent critical junctures in agricultural evolution. The initial benefits of HR crops—simplified weed control and enhanced crop productivity—are now balanced against the complexities of managing herbicide resistance among weed populations. This balance calls for a shift towards more sustainable and integrated approaches to weed management. Looking ahead, the integration of novel herbicide discoveries and genetic targets for herbicide resistance into traditional agricultural practices holds considerable promise. The exploration of natural products and the application of advanced molecular techniques are leading to the identification of new herbicidal MOAs and resistance mechanisms. This progress is crucial for staying ahead of the adaptive weed species that threaten crop yields globally.

SSWM is emerging as a pivotal technology in precision agriculture, offering the potential to drastically reduce herbicide use while enhancing the specificity and effectiveness of applications. The development and refinement of SSWM technologies, alongside the implementation of cutting-edge sensors and AI-driven decision-making tools, are vital to making these systems practical for widespread adoption. Moreover, the resurgence of non-chemical control strategies, such as HWSC and the breeding of competitive cultivars, is diversifying the weed management toolkit. These methods lessen dependence on chemical controls and help prevent further resistance developments.

However, the integration of HR crops, novel herbicides, and comprehensive IWM faces significant regulatory, economic, and social challenges. Regulatory frameworks need to support innovative agricultural technologies while promoting sustainable usage. Policymakers must work with scientists, farmers, and industry stakeholders to create incentives for adopting sustainable practices and ensure regulations are scientifically grounded and adaptive. Ongoing research into HR crop development, novel herbicide discoveries, and alternative weed management strategies is essential. Improving genetic engineering techniques and expanding our understanding of weed biology and ecology will support these efforts. Moreover, linking technological innovations directly with ecological stewardship will align agricultural practices with environmental sustainability.

In conclusion, an integrated approach that merges technological innovation with ecological sensitivity is the most promising route to sustaining global crop productivity and resilience. By continuously adapting our strategies to the evolving dynamics of weed populations and agricultural ecosystems, we can ensure the long-term sustainability and efficiency of our agricultural practices.

## Data Availability

No datasets were generated or analysed during the current study.

## Abbreviations

HR: Herbicide-resistant
IWM: Integrated weed management
HWSC: Harvest weed seed control
SSWM: Site-specific weed management
ALS: Acetolactate synthase
ACCase: Acetyl-CoA carboxylase
EPSPS: 5-Enolpyruvyl shikimate-3-phosphate synthase
MOAs: Modes of action
HPPD: Hydroxyphenylpyruvate dioxygenase
PAT: Phosphinothricin acetyltransferase
GMOs: Genetically modified organisms
PRE: Preemergence
POST: Postemergence
MAS: Marker-assisted selection
GWAS: Genome-wide association studies
VRT: Variable rate technology
AI: Artificial intelligence
